# Suppression of miR-221 inhibits glioma cells proliferation and invasion via targeting SEMA3B

**DOI:** 10.1186/s40659-015-0030-y

**Published:** 2015-07-22

**Authors:** Guilan Cai, Shanshan Qiao, Kui Chen

**Affiliations:** Department of Neurology, Beijing Friendship Hospital, Capital Medical University, 95 Yong’an Rd, Xicheng, Beijing, 100050 China

**Keywords:** miR-221, Glioma, SEMA3B, Cancer metastasis

## Abstract

**Background:**

Gliomas are the most common primary tumors in the central nervous system. Due to complicated signaling pathways involved in glioma progression, effective targets for treatment and biomarkers for prognosis prediction are still scant.

**Results:**

In this study we revealed that a new microRNA (miR), the miR-221, was highly expressed in the glioma cells, and suppression of miR-221 resulted in decreased cellular proliferation, migration, and invasion in glioma cells. Mechanistic experiments validated that miR-221 participates in regulating glioma cells proliferation and invasion via suppression of a direct target gene, the Semaphorin 3B (*SEMA3B*). The rescue experiment with miR-221 and SEMA3B both knockdown results in significant reversion of miR-221 induced phenotypes.

**Conclusion:**

Taken together, our findings highlight an unappreciated role for miR-221 and SEMA3B in glioma.

## Background

Glioma is one of the major human primary brain tumors [1], which is characterized by tumor cell local invasion and migration from remote distances [2]. Though significant progress has been made in surgical and chemotherapy treatment, the prognosis of patients with glioma remains poor [3]. Therefore, new therapeutic strategies for the management of glioma is therefore essential.

MicroRNAs (miRNAs) are endogenous small non-coding RNAs of about 22 nucleotides in length, which regulate gene expression post-transcriptionally. MiRNAs recognize and repress target mRNAs based on sequence complementarity between the miRNA and the target mRNA, and are critical in regulating a variety of biological processes, including cell cycle, differentiation, development, and metabolism, as well as diseases such as diabetes, neurodegenerative disorders, and cancers [4]. Emerging literatures have found that aberrant miRNAs could act as oncogenes or tumor suppressors in glioma tumorigenesis [5]. Dysregulation of these miRNAs contributes to tumorigenesis by stimulating glioma cells proliferation, angiogenesis and invasion [6, 7]. Among these miRNAs the miR-221/222 are attractive to researchers because of their bimodal function in the tumorigenesis of human cancers [8]. They are encoded in tandem from a gene cluster located on chromosome X, and both tumor-suppressive and oncogenic roles of miR-221/222 have been reported in a series of cancer types [9]. A couple of up-to-date studies have revealed collaborations between the expression of miR-221/222 and glioma carcinogenesis, since higher miR-221/222 expression correlated with poorer prognostic status of patients, which might be attributed to their attenuated sensitivity to chemotherapy and radiotherapy [10]; in addition Zhang et al. [11] reported plasma miR-221/222 family levels were significantly upregulated in glioma patients, and high positive plasma miR-221 and miR-222 were both correlated with poor survival rate. These studies suggested that detection of the miRNA-221/222 family should be considered as a new additional tool to better characterize glioma diagnostic and predictive value of miR-221/222 family for glioma. However, functions of miR-221/222 in glioma tumorigenesis and development are poorly understood.

In this study, we aimed at miR-221 as a candidate miRNA according to the RT-qPCR screening and validated its expression in glioma tissues and cell lines. Further researches to assess the effects of miR-221 on glioma cells proliferation and invasion were carried on, as well as the regulating mechanism on *SEMA3B* gene was explored.

## Results

### miR-221 expression was elevated in glioma cells

**Figure 1:**
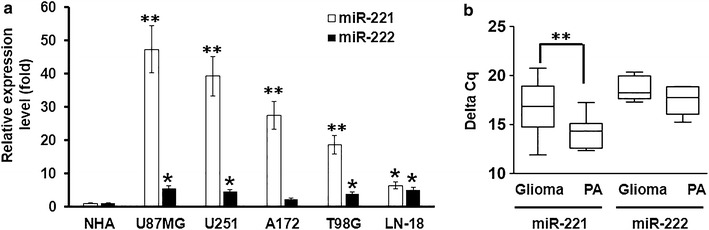
miR-221 expression in human glioma cells and glioma tissues from patients. **a** RT-qPCR showed the measurement of miR-221 and miR-222 expressions in different human glioma cell lines. **b** RT-qPCR assay showed the measurement of miR-221 and miR-222 expressions in 30 glioma tissues or paracarcinoma tissues from patients. GAPDH serves as the endogenous control. *p < 0.05; **p < 0.01.

We aimed to explore the potential functions and molecular mechanisms of miR-221/222 family in glioma. The U87MG, U251, A172, T98G and LN-18 glioma cell lines and the normal human astrocytes cell line NHA were chosen for further research. The expression of miR-221 and miR-222 were determined by qPCR, the result showed that miR-221 was greatly upregulated in glioma cells, while miR-222 was only slightly upregulated (Figure 1a). Furthermore, a total of 30 cases of human glioma samples were chosen to detect the miR-221/222 expression levels in glioma tissues compared with the paracancerous tissue, respectively. Interestingly, our data showed that the only expression of miR-221 between the glioma group and the paraglioma group showed significant increasing, but miR-222 expression had no obvious difference (Figure 1b).

### Suppression of miR-221 inhibits cell growth in glioma cells

**Figure 2:**
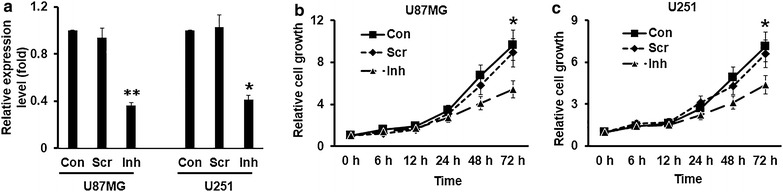
Suppression of miR-221 inhibits cell growth in U87MG and U251 glioma cells. **a** RT-qPCR was performed to measure miR-221 expression levels in loss of function model. *Scr* scramble, *Inh* miR-221 inhibitor. CCK-8 assay was employed to detect the cell proliferation in U87MG cells (**b**) and U251 cells (**c**). Glioma cells were treated with 100 pM of miR-221 inhibitors for 48 h and then cells were replaced into a 96-well plate for up to 72 h, and cells were collected for assigned time points for CCK-8 assay *p < 0.05; **p < 0.01.

To examine the function of miR-221, we firstly silenced the expression of miR-221 using the miR-221 inhibitor in glioma cells. After the U87MG and U251 cells were treated with 100 pM of miR-221 for 48 h, we employed the qPCR to detect the expression level of miR-221, and the result showed that miR-221 was significantly reduced both in U87MG and in U251 cells (Figure 2a). Then we used these glioma cells with miR-221 knockdown to test whether miR-221 participates in the cell growth of glioma cells. After treated with miR-221 inhibitor (100 pM) in U87MG and U251 cells for 48 h, we replated these cells in a 96-well plate and the CCK-8 assay was performed to verify the glioma cell proliferation up to 3 days. Our results showed that the proliferation rate was gradually decreased in cells transfected with miR-221 inhibitor compared with the media control and the scramble control; significant difference was observed at 72 h time point in both U87MG cells and U251 cells (Figure 2b, c). These data indicated that miR-221 participated in the proliferation of glioma cells and suppression of miR-221 could inhibit cell growth.

### Suppression of miR-221 inhibits glioma cells migration and invasion

**Figure 3:**
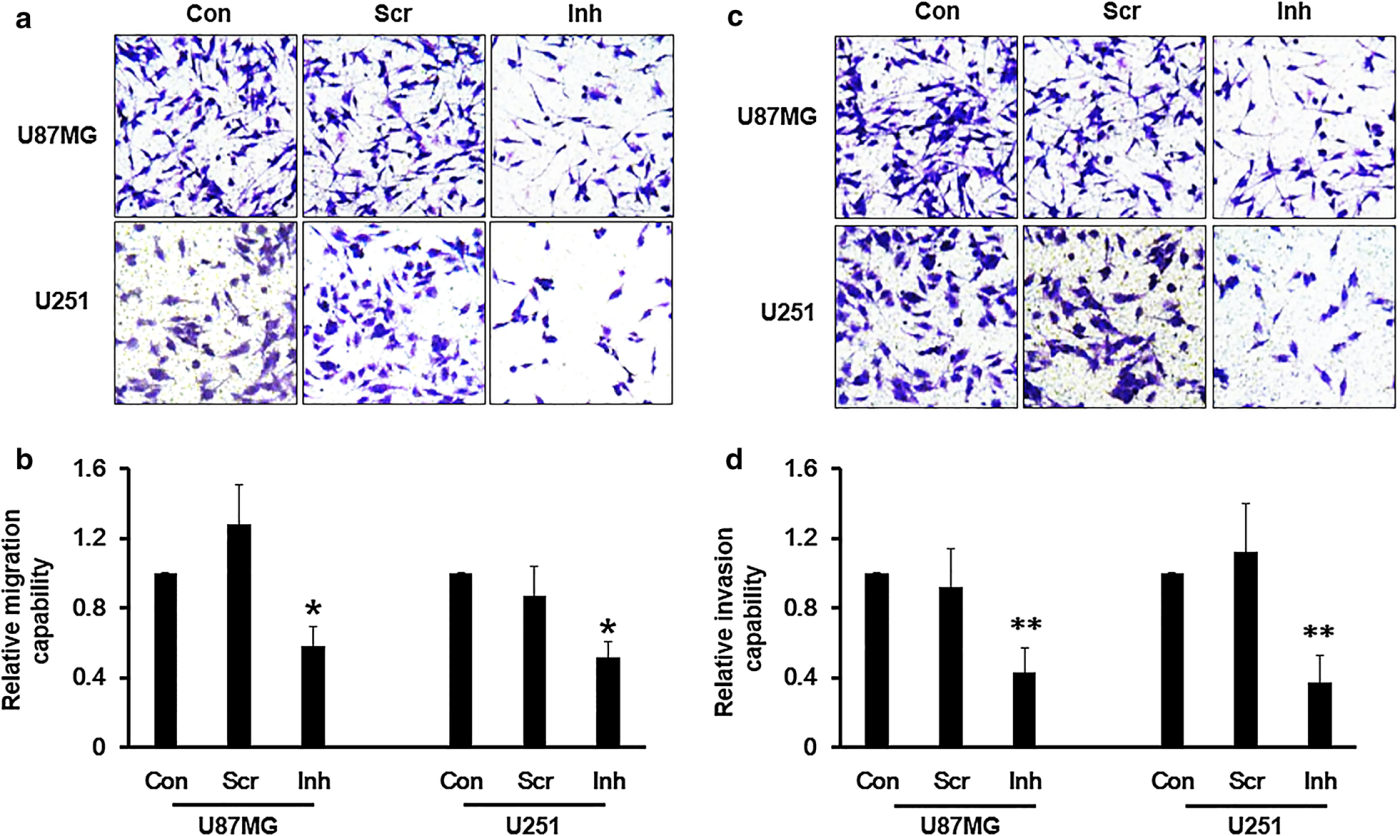
Suppression of miR-221 inhibits migration and invasion in glioma cells. **a** Photography and **b** quantitative assay of the transwell assay without matrigel coated was employed to detect the cell migration ability in U87MG and U251 cells with miR-221 knockdown using miR-221 inhibitor. **c** Photography and **d** quantitative assay of the transwell assay with matrigel coated was employed to detect the cell invasion ability in U87MG and U251 cells with miR-221 knockdown. *p < 0.05; **p < 0.01.

Glioma cells exerts powerful migration and invasion, and plenty of genes and micro RNAs have been revealed in contribution to the metastasis. We hypothesized that high expression level of miR-221 in glioma cells might be involved in this pathologic process in glioma cells. To study whether miR-221 indeed participates in the invasion, we performed transwell assay without matrigel to detect the migration ability and transwell assay with matrigel to verify the invasion ability. As shown in the Figure 3, compared with the media control and the scramble control, the miR-221 inhibitor treated U87MG and U251 cells showed significant lower rates both in migration ability (Figure 3a, b) and invasion ability (Figure 3c, d), both were clearly shown in the photography and quantitative analysis.

### SEMA3B is the target for miR-221

**Figure 4:**
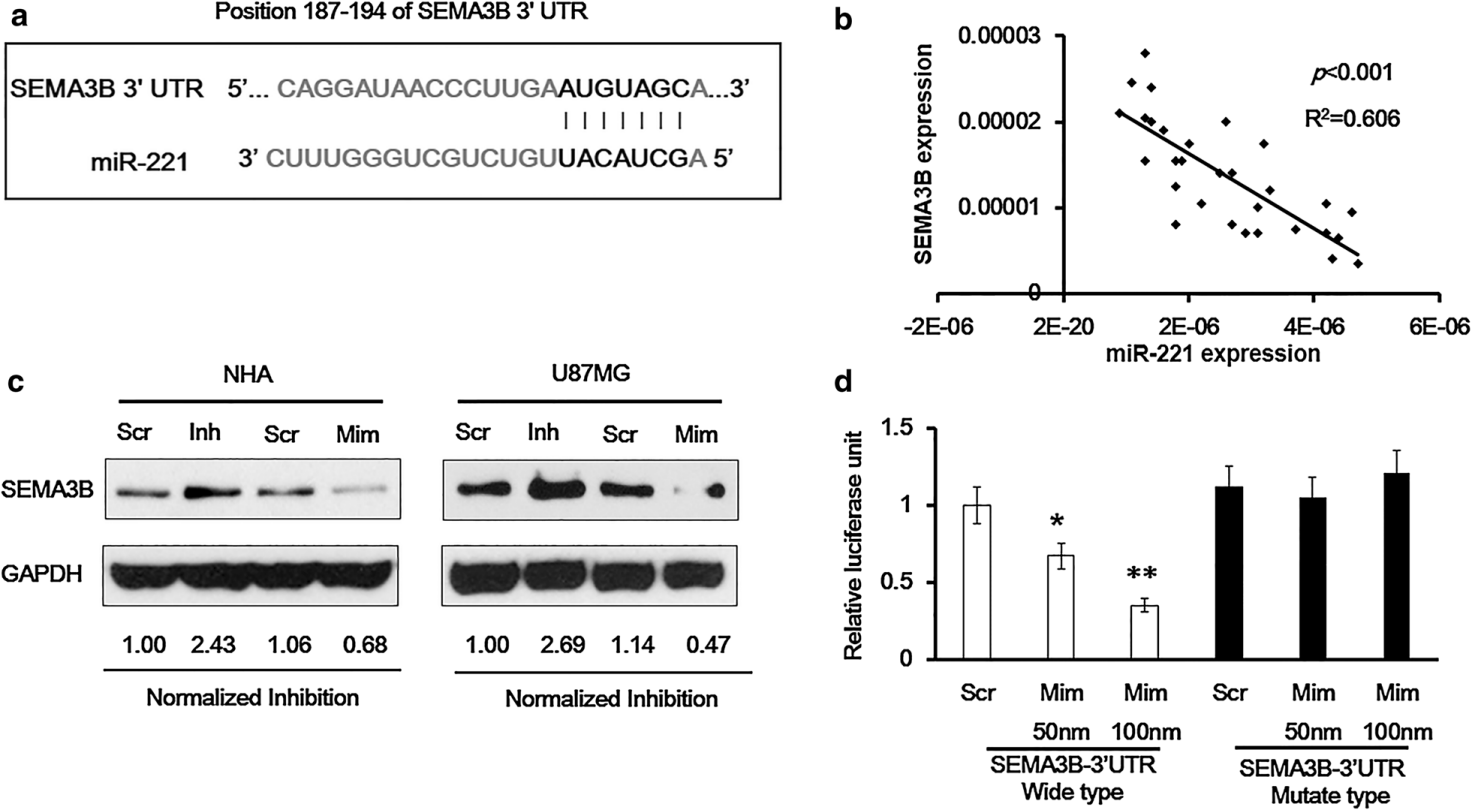
SEMA3B is a target of miR-221. **a** Binding sites of miR-221 (the *black letters*) on the SEMA3B mRNA 3′UTR. **b** An inverse correlation between miR-221 and SEMA3B was observed by Spearman correlation analysis in 30 cased of human glioma tissues. **c** Western blot was performed to detect the protein level of SEMA3B in NHA and U87MG cells with miR-221 overexpression or knockdown using miR-221 inhibitor or mimic (100 pM) for 48 h, respectively. **d** Luciferase reporter plasmids and miR-221 mimics were co-transfected to cells, and then the luciferase reporter assay was employed to detect luciferase activity. *p < 0.05; **p < 0.01.

To elucidate the detailed mechanisms of miR-221 in regulating glioma cell biology, we predicted its potential targets using TargetScan (http://www.targetscan.org). We found that the Semaphorin 3B gene (SEMA3B) is a theoretical target of miR-221, and there are seven miR-221 conserved binding sites on the 3′ UTR region of SEMA3B mRNA (Figure 4a). Most importantly, SEMA3B regulates neuronal migration and acts as a tumor a suppressor gene [12]. We firstly analyzed the correlation of the expression of miR-221 and SEMA3B level in 30 human glioma samples using RT-qPCR, and out result presented that there was a negative correlation between miR-221 and SEMA3B (Figure 4b). We manipulated the expression of miR-221 using mimic or inhibitor both in the Normal Human Astrocytes (NHA) and human glioma U87MG cells, and the SEMA3B level was detected using western blot. We found that although miR-221 inhibitor could upregulate the protein level of SEMA3B, and miR-221 mimic could downregulate expression of SEMA3B both in the NHA cells and in the U87MG cells, respectively, the regulating degree was much more significant in the U87MG cells (Figure 4c). In addition, we used luciferase assay reporters containing the wild type 3′UTR sequence and the mutant type (with 3 of the miR-221 binding sites changed) of SEMA3B mRNA to test the effect of miR-221 on the transcriptional activity. Relative luciferase activity upon transfection of scramble control (Scr) or different dosage of miR-221 mimic (50 and 100 pM) demonstrated that only the miR-152 mimic, but not the scramble, suppressed transcriptional activity of the 3′UTR of SEMA3B in a dose dependent manner (Figure 4d, left panel). On the other hand, in the mutant vector, inhibitory effect of miR-221 mimic on the 3′UTR of SEMA3B mRNA was not observed (Figure 4d, right panel).

### Knockdown endogenous SEMA3B reversed the effect of miR-221 Inhibitor

**Figure 5:**
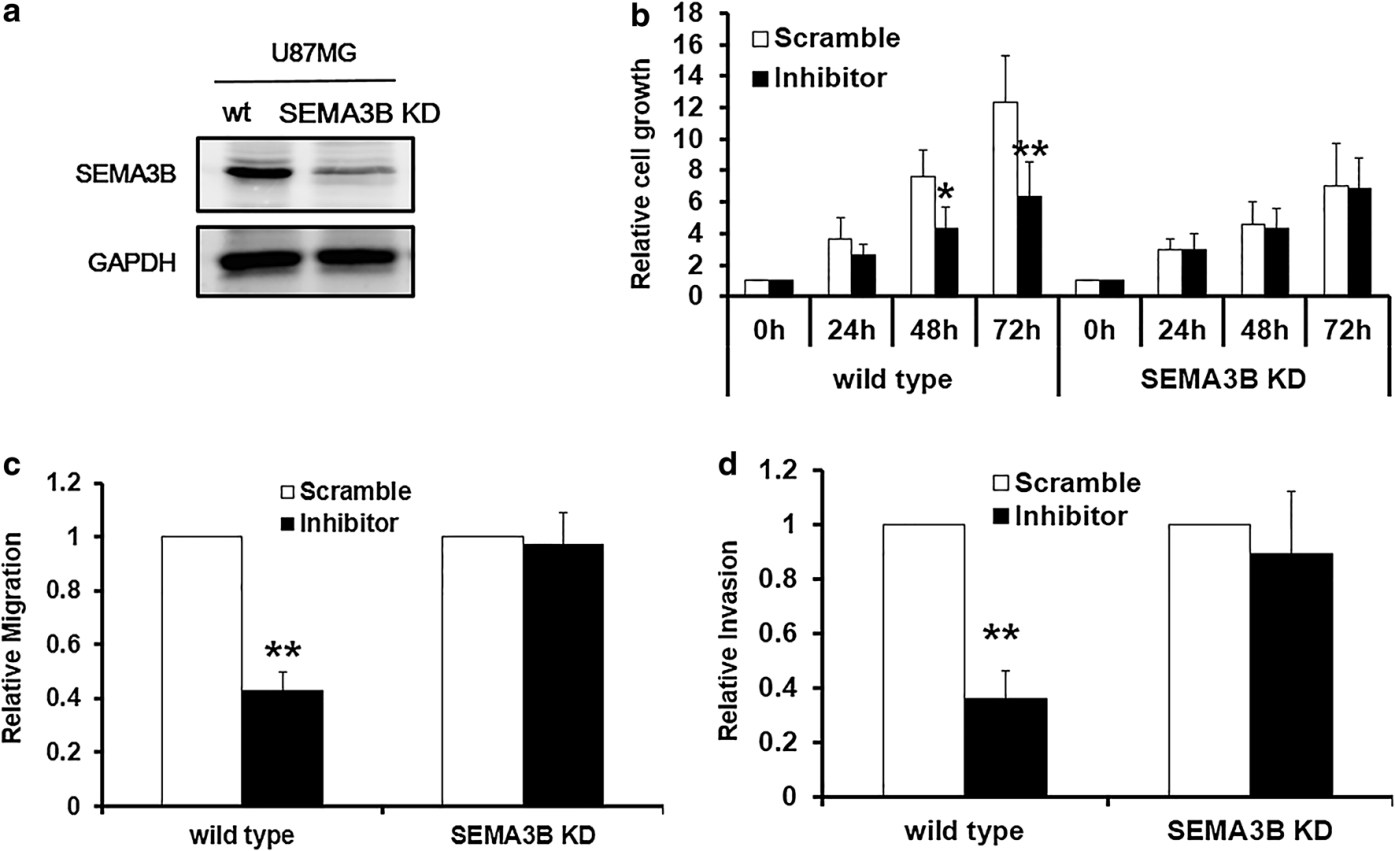
Knockdown endogenous SEMA3B reversed the effect of miR-221 Inhibitor. **a** Western blot showed SEMA3B level in a U87MG cells with SEMA3B gene stably silenced, **b** CCK-8 assay was employed to detect the cell proliferation in U87MG wild type and SEMA3B KD cells treated with scramble or miR-221 inhibitor (100 pM). Transwell assay without matrigel coated for testing cell migration (**c**) and transwell assay with matrigel coated was employed to detect the cell invasion ability (**d**) in U87MG cells treated with scramble or miR-221 inhibitor (100 pM). *p < 0.05; **p < 0.01.

In order to confirm whether miR-221 indeed exerts its function through its target SEMA3B, we established a U87MG cell line in which the SEMA3B gene was stably silenced (SEMA3B KD) by SEMA3B shRNA (Figure 5a). miR-221 inhibitor and scramble control were transfected into the wild type U87MG cells and the SEMA3B KD U87MG cells, and CCK-8 assay and transwell assay were employed respectively to test the cell proliferation, migration, and invasion ability changes. The results demonstrated that the miR-221 inhibitor could only suppress the cell proliferation in the wild type U87MG cells but not in the SEMA3B cell (Figure 5b). Inhibitory effect of miR-221 on U87MG cell invasion and invasion were only observed in the wild type U87MG cells as expected (Figure 5c, d). These data suggest that miR-221 mediated cell growth and invasion through its transcriptional modulation on SEMA3B.

## Discussion

Glioma is the most general primary tumor in nervous system with the highest mortality and mortality among endocranial tumors for its forceful malignant proliferation and invasion [13, 14]. An early diagnosis of glioma in patients greatly increases the chances for successful treatment, therefore a specific and sensitive marker is very important. Previous studies for identifying glioma biomarkers of glioma had mainly focused on proteins [15], however, miRNAs have absorbed more and more of attention from researchers for the advantage of easy and low cost of determination as new molecular markers [16]. The aim of this study is to evaluate the function of miR-221 in human glioma and the potential as diagnostic and treatment marker. In the current study, we have found that miR-221 level in glioma is significantly higher than that in non-glioma tissues or cells; that miR-221 participates in glioma cell proliferation, migration and invasion; that miR-221 plays its biological roles via negatively regulating the SEMA3B gene in glioma. All the results suggested that miR-221 could be used as potential biomarkers in glioma identification, early diagnosis, and developing new therapeutic strategies.

Semaphorins families are a large number of cytoplasmic and membrane-bound proteins, including SEMA1–A4, B1–B3, and C1 in vertebrate [17]. The SEMA3B gene is widely expressed in the mammary gland, colon, lung, kidney, and so on [18]. The SEMA3B protein is an antagonist of receptors for neuropilins 1 and 2 (Np1 and Np2) that also act as receptors for several isoforms of vascular endothelial growth factor (VEGF), which is a general initiator of tumor angiogenesis, and thus SEMA3B suppresses vascular growth in tumors [19]. Suppression in transcriptional activity of this gene in tumors indicate that SEMA3B may participate in cell growth suppression in the kidney, ovary, and colon cancers [20]. In our study, the expression level of SEMA3B in glioma cells was found lower than normal glia cells, which is the first observation of SEMA3B in glioma cells to our knowledge and further result suggest that SEMA3B participates in the regulation of glioma cells proliferation and invasion. Though SEMA3B was verified for the target of miR-221 by us for the first time, there were still some other targets of miR-221 existence, which needed us to explore in future.

Mounting evidence implicates miRNAs as regulators of the tumor phenotype through their ability to modulate the expression of critical genes and signaling networks involved in tumorigenesis and downstream malignant processes [21]. miR-221/222 act as oncogenic miRNAs that facilitate cell proliferation via down-regulation of p27Kip1 and/or p57Kip2, which negatively regulate cell cycle progression from G1- to S-phase [22–24]. Although various studies present the mechanism by which miR-221 functions in cancer cells, the oncogene or tumor suppression capability of miR-221 in glioma was poorly understood since there were only few papers before this study. Zhang et al. [25] demonstrated that high levels of miR-221/222 expression in gliomas confer highly aggressive invasion and poorer overall survival through by targeting TIMP3 and act as prognostic factors for glioma patients; Cristina et al. reported that overexpression of miR-221/222 produces an increase in sensitivity to temozolomide via a reduction in the level of MGMT. In addition, these miRNAs increase DNA damage, conferring oncogenic features to glioma cells [26]. In this study, we found miR-221, but not miR-222 level is significantly high in the glioma tissues and cells, and manipulating of expression of miR-221 could result in glioma cell proliferation, migration and invasion ability change, indicating that miR-221 is a critical oncogene in glioma. However, exploring the detailed function of miR-221/222 and searching more targets of miR-221/222 in glioma cells are the problems to be solved.

## Conclusions

In summary, we described that miR-221 expression level is associated with glioma tumorigenesis. The loss of function of miR-221 in glioma cell line suggests that knockdown of miR-221 inhibits cell proliferation and invasion. Mechanistically, SEMA3B is the direct target of miR-221, which acts as the tumor suppressor in glioma. These results suggest that miR-221 plays an important role in glioma tumorigenesis and could be served as a potential molecular marker for the diagnostic and treatment of glioma.

## Methods

### Experimental subjects

There were totally 102 patients involved at the initiation of this study in the Department of neurology, Beijing Friendship Hospital, Capital Medical University, and only 30 cases with surgical resection of brain glioma were collected according to the strict inclusive criteria: (1) no other types of tumor, viral hepatitis, or autoimmune diseases; (2) no preoperative chemotherapy or radiation therapy. The paracancerous tissue was defined as 2 cm away from lesions. All specimens were obtained under sterile conditions during surgery. All patients were given informed consent, and this study was approved by the Ethic Committee Board of Capital Medical University.

### Cell preparation and culture

NHA cells were purchased from Lonza Company (CA, USA), U87MG, U251, A172, T98G and LN-18 glioma cell lines were purchased from ATCC Company (VA, USA). All the cells were maintained in DMEM medium with high glucose and sodium pyruvate, supplemented with 10% fetal bovine serum and antibiotics (100 units/ml penicillin and 100 mg/ml streptomycin). Cells were incubated at 37°C in a humidified atmosphere of 5% CO_2_ in air.

### Transfection of shRNA

The shRNA directed against Sema3b and the control shRNA were both chemically synthesized, the sequence of shRNA used for Sema3b were Sense: 5′-GGCCGACUGUACUAAAGUAUU-3′, Antisense: 5′-UACUUUAGUACAGUCGGCCUU-3′; the sequence of control shRNA were Sense: 5′-GAUAGACAAAUGACGAAUGCGUAUU-3′, Antisense: 5′-TCGCTTCGTCTTTGTCTTCUU-3′. When the cells reached 70% confluent, Oligofectamine reagent from Invitrogen (Life Technology, NY, USA) was employed to perform the in vitro transfection according to the manufacturer’s instructions, and cells were harvested 48 h after transfection for further experiments.

### CCK-8 assay

Cells were seeded in 96-well plates at confluence of 2,000 cells per well. The absorptions of the cells were measured using CCK-8 kit (Dojindo Laboratories, Japan) based on the manufacturer’s instruction at indicated time points.

### Transwell assay

Transwell membranes were coated with matrigel for 6 h for invasion assay, or without matrigel for migration assay. 5 × 10^4^ cells were seeded onto the upper chamber, and 800 μl medium with 10% fetal bovine serum was added to the lower chamber. After incubation for 24 h, cells adhering to the upper surface of the membrane were removed with a cotton swab. The invasion or migration cells, which adhered to the lower surface, were fixed with 4% paraformaldehyde and stained with 0.1% crystal violet. The cells were finally extracted with 33% acetic acid and detected quantitatively using microplate reader (at 570 nm). Data were obtained from three independent experiments.

### Quantitative real-time PCR (qPCR)

RNAs were isolated using an RNAvzol reagent (Vigorous Biotechnology, Beijing, China) according to the manufacturer’s protocol. cDNA was synthesized with M-MLV reverse transcriptase (Promega, WI, USA) in a 20 μl reaction mixture (2 µg total RNA, 2 µl 10 × RT buffer, 1 µl M-MLV enzyme, 1 µl dNTP, 1 µl miR primer, 1 µl random primer, and ddH_2_O to 20 µl) at 37°C for 2 h. RT-qPCR was performed in the ABI 7500 real-time RT-PCR system with reagents from the SYBR^®^ Green Real-time PCR Master Mix (TOYOBO, Japan) and the appropriate primers in a 20 µl reaction system (10 µl Master mixture, 1 µl cDNA, 1 µl forward primer and 1 µl reverse primer, and 7 µl ddH_2_O). A 10 min “hot start” was carried on firstly, and then 40 cycles were run with each cycle including a denaturation step at 95°C for 10 s, annealing starting at 60°C for 20 s, and amplification at 72°C for 30 s. For quantification of the target gene, the ΔΔCt algorithm was used. The primers for miR-221, miR-222 and U6 were all purchased from RiboBio Company. All experiments were performed at least three times and the mean values were used.

### Western blot analysis

Cells were washed twice in ice-cold PBS and lysed in 1 × lysis buffer (Cell Signal Technology, CA, USA), the protein concentration of each sample was detected using BCA assay before loading, and then whole cell extracts (50 μg protein) was analyzed by 12% SDS PAGE and subsequently transferred onto nitrocellulose membranes. For immunoblot experiments, membranes were blocked for 1 h with 5% skimmed milk powder in TBS containing 0.1% Tween-20, and incubated with primary antibody at 4°C overnight. The rabbit polyclonal anti-SEMA3B antibody (code: sc-21204-R, 1:500 dilution) and the goat polyclonal anti-GAPDH antibody (code: sc-48167, 1:2,000 dilution) were both purchased from Santa Cruz Biotechnologies (CA, USA). The secondary antibodies were all purchased from Zhongshanjinqiao Company (Beijing, China), bound antibodies were visualized with an enhanced chemiluminescence system (DuPont, Boston, MA). The densitometric analysis was been carried on using the ImageJ software (Fiji Software), and the percentage of inhibition was normalized as SEMA3B/GAPDH.

### Luciferase reporter assay

The luciferase reporter containing the 3′ UTR of the human SEMA3B gene was purchased from RiboBio Company (Guangzhou, China). HeLa cells were co-transfected with miR-221 mimics (100 pM) with luciferase reporter for 48 h, and Renilla luciferase expression construct. Cells were harvested 36 h post-transfection and assayed with Dual Luciferase Assay (Promega, WI, USA) according to the manufacturer’s instructions. Luciferase activity was measured and normalized to Renilla luciferase activity. Three independent experiments were performed in triplicate.

### Statistical analysis

The data are expressed as mean ± standard deviation (SD). Comparisons between groups were analyzed using Student’s t test or ANOVA. Differences were considered to be statistically significant at *p* < 0.05.
